# Bis[4-oxido-2-oxo-2,3-di­hydro­pyrimidin-1-ium-5-carboxyl­ato(1.5−)-κ^2^ *O* ^4^,*O* ^5^]bis­(1,10-phenanthroline-κ^2^ *N*,*N*′)dysprosium(III) dihydrate

**DOI:** 10.1107/S1600536809036848

**Published:** 2009-09-19

**Authors:** Xianlin Liu, Huihui Xing, Jing Miao, Maomao Jia, Zilu Chen

**Affiliations:** a College of Chemistry and Chemical Engineering, Guangxi Normal University, Yucai Road 15, Guilin 541004, People’s Republic of China

## Abstract

In the title compound, [Dy(C_5_H_2.5_N_2_O_4_)_2_(C_12_H_8_N_2_)_2_]·2H_2_O, the Dy^III^ ion is located on a twofold rotation axis and is coordinated in a square-anti­prismatic geometry by two chelating 1,10-phenanthroline mol­ecules as well as two 4-oxido-2-oxo-2,3-di­hydro­pyrimidin-1-ium-5-carboxyl­ato(1.5−) anions. N—H⋯O and O—H⋯O hydrogen bonds help to stabilize the crystal structure. The H atom involved in an N—H⋯N hydrogen bond is disordered around a twofold rotation axis.

## Related literature

For isostructural lanthanide complexes with 2,4-dioxo-1,2,3,4-tetra­hydro­pyrimidine-5-carboxylic acid, see: Sun & Jin (2004*a* ▶,*b* ▶); Xing *et al.* (2008*a* ▶); Xiong *et al.* (2008*a* ▶,*b* ▶). For other metal complexes of 2,4-dioxo-1,2,3,4-tetrahydropyrimidine-5-carboxylic acid, see: Hueso-Ureña *et al.* (1993 ▶, 1996 ▶); Baran *et al.* (1996 ▶); Luo *et al.* (2002 ▶); Maistralis *et al.* (1991 ▶, 1992 ▶); Xing *et al.* (2008*b* ▶). For the role played by hydrogen bonds in stabilizing structures, see: Chen *et al.* (2006 ▶); Arora *et al.* (2009 ▶); Jagan & Sivakumar (2009 ▶).
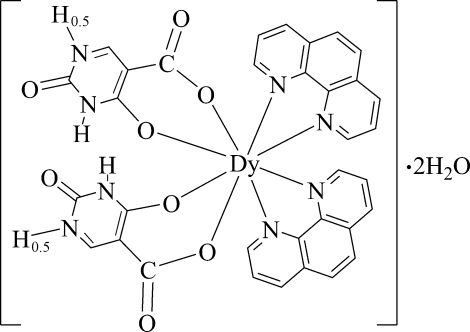


## Experimental

### 

#### Crystal data

[Dy(C_5_H_2.5_N_2_O_4_)_2_(C_12_H_8_N_2_)_2_]·2H_2_O
*M* *_r_* = 868.12
Monoclinic, 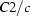

*a* = 17.143 (2) Å
*b* = 14.4470 (17) Å
*c* = 13.2211 (16) Å
β = 100.613 (2)°
*V* = 3218.3 (7) Å^3^
*Z* = 4
Mo *K*α radiation
μ = 2.40 mm^−1^
*T* = 295 K
0.10 × 0.03 × 0.02 mm

#### Data collection

Bruker APEXII CCD area-detector diffractometer
Absorption correction: multi-scan (*SADABS*; Bruker, 1998 ▶) *T*_min_ = 0.796, *T*_max_ = 0.954
11116 measured reflections
2842 independent reflections
2619 reflections with *I* > 2σ(*I*)
*R*_int_ = 0.037

#### Refinement

*R*[*F* ^2^ > 2σ(*F* ^2^)] = 0.030
*wR*(*F* ^2^) = 0.068
*S* = 1.06
2842 reflections
240 parameters
H-atom parameters constrained
Δρ_max_ = 0.93 e Å^−3^
Δρ_min_ = −1.22 e Å^−3^

### 

Data collection: *APEX2* (Bruker, 2004 ▶); cell refinement: *SAINT* (Bruker, 2004 ▶); data reduction: *SAINT*; program(s) used to solve structure: *SHELXS97* (Sheldrick, 2008 ▶); program(s) used to refine structure: *SHELXL97* (Sheldrick, 2008 ▶); molecular graphics: *SHELXTL* (Sheldrick, 2008 ▶); software used to prepare material for publication: *SHELXTL*.

## Supplementary Material

Crystal structure: contains datablocks global, I. DOI: 10.1107/S1600536809036848/pb2007sup1.cif

Structure factors: contains datablocks I. DOI: 10.1107/S1600536809036848/pb2007Isup2.hkl

Additional supplementary materials:  crystallographic information; 3D view; checkCIF report

## Figures and Tables

**Table 1:** Selected geometric parameters (Å, °)

Dy—O2	2.256 (3)
Dy—O1	2.313 (2)
Dy—N2	2.554 (3)
Dy—N1	2.576 (3)

**Table 2:** Hydrogen-bond geometry (Å, °)

*D*—H⋯*A*	*D*—H	H⋯*A*	*D*⋯*A*	*D*—H⋯*A*
N3—H3⋯N3^ii^	0.86	1.80	2.659 (7)	174
N4—H4⋯O3^iii^	0.86	2.01	2.867 (4)	178
N4—H4⋯O2^iii^	0.86	2.62	3.183 (4)	124
O5—H51⋯O3^iv^	0.85	2.15	2.993 (5)	175
O5—H52⋯O4^v^	0.85	2.15	2.960 (5)	160

Symmetry codes: (ii) 
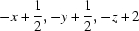
; (iii) 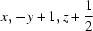; (iv) 
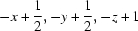
; (v) 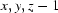.

**Fig. 1.**
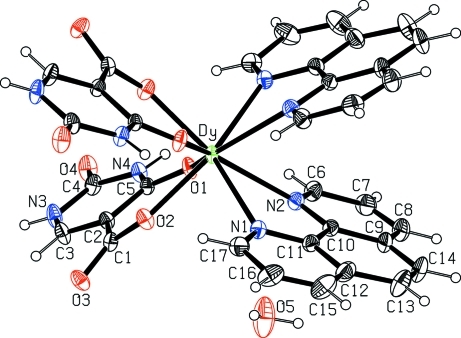
The molecular structure of the title compound with the atom-numbering scheme and 30% displacement ellipsoids.

**Fig. 2.**
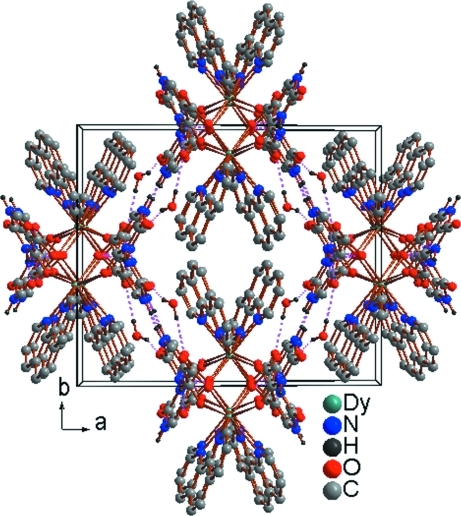
A view of the packing diagram of the title compound viewed down the c axis. H atoms not involving in hydrogen bonds are omitted for clarity.

## supplementary crystallographic information

### Comment

2,4-Dioxo-1,2,3,4-tetrahydropyrimidine-5-carboxylic acid has been extensively used in the preparation of robust networks or some porous coordination polymers because of its versatile coordination modes. For further investigation of its coordination behavior to lanthanide(III) ions, we report here a new Dy^III^ complex of 2,4-dioxo-1,2,3,4-tetrahydropyrimidine-5-carboxylic acid, which turned out to be isostructural with the analogues Eu^III^, Tb^III^, Yb^III^ (2004*a*,*b*), Er^III^ (Xing *et al.*, 2008*a*) and Y^III^, Gd^III^ (Xiong *et al.*, 2008*a*,*b*) complexes.

In the title complex, (I), the Dy^III^ ion is located on a twofold rotation axis and coordinated in square-antiprismatic geometry completed by four N atoms from two chelating phenanthroline molecules, four O atoms from two chelating 4-oxido-2-oxo-2,3-dihydropyrimidin-1-ium-5-carboxylate(1.5-) anions. The distances of Dy—O are 2.256 (3) and 2.313 (2)Å, and those of Dy—N are 2.554 (3) and 2.576 (3)Å. The averge distance of Dy—O is 2.285Å, which is much shorter than that of Dy—N (2.565Å). The dihedral angle between two phen molecules is 37.726°. As shown in Table 2 and Fig. 2, each coordination unit [Dy(C_5_H_3_N_2_O_4_)(C_5_H_2_N_2_O_4_)(C_12_H_8_N_2_)_2_)] interacts with another four *via* intermolecular N—H···O and N—H···N hydrogen bonds. Each lattice water molecule links two coordination units mentioned above *via* O—H···O hydrogen bonds. As a result, these hydrogen bonds link the title complex units and lattice water molecules into a three-dimensional supramolecular network as shown in the packing diagram (Fig. 2). The H atom involved in an N—H···N hydrogen bond is disordered around a twofold rotation axis with half occupancy.

### Experimental

A mixture of 2,4-dioxo-1,2,3,4-tetrahydropyrimidine-5-carboxylic acid (0.0312 g, 0.2 mmol), DyCl_3_.6H_2_O (0.0754 g, 0.2 mmol), Phen.H_2_O (0.0396 g, 0.2 mmol), NaOH (0.00800 g, 0.2 mmol), water (15 ml) was sealed in a 25 ml,Teflon-lined stainless-steel reactor and heated to 383 K for 120 h, it was then cooled over 48 h, light yellow crystals were isolated in 80% yield. Elemental analysis for C_34_H_25_DyN_8_O_10_, calculated: C 47.04, H 2.90, N 12.91%; found: C 47.49, H 2.96, N 13.24%.

### Refinement

H atoms of the water molecule were located in a difference Fourier map and allowed to ride on their parent atoms [*U*_iso_(H) = 1.5*U*_eq_(O)]. Other H atoms were placed at calculated positions (C–H = 0.93 Å and N—H = 0.86 Å) and were included in the refinement in the riding model approximation, with *U*_iso_(H) = 1.2*U*_eq_(C, N). The pyrimidine hydrogen atom H3 is shared by two N—H groups and thus has an occupancy factor of 0.5.

### Crystal data

**Table:**

[Dy(C_5_H_2.5_N_2_O_4_)_2_(C_12_H_8_N_2_)_2_]·2H_2_O	*F*(000) = 1724
*M**_r_* = 868.12	*D*_x_ = 1.792 Mg m^−^^3^
Monoclinic, *C*2/*c*	Mo *K*α radiation, λ = 0.71073 Å
Hall symbol: -C 2yc	Cell parameters from 4430 reflections
*a* = 17.143 (2) Å	θ = 2.3–26.9°
*b* = 14.4470 (17) Å	µ = 2.40 mm^−^^1^
*c* = 13.2211 (16) Å	*T* = 295 K
β = 100.613 (2)°	Prism, yellow
*V* = 3218.3 (7) Å^3^	0.10 × 0.03 × 0.02 mm
*Z* = 4	

### Data collection

**Table:**

Bruker APEXII CCD area-detector diffractometer	2842 independent reflections
Radiation source: fine-focus sealed tube	2619 reflections with *I* > 2σ(*I*)
graphite	*R*_int_ = 0.037
φ and ω scans	θ_max_ = 25.0°, θ_min_ = 1.9°
Absorption correction: multi-scan (*SADABS*; Bruker, 1998)	*h* = −20→20
*T*_min_ = 0.796, *T*_max_ = 0.954	*k* = −17→17
11116 measured reflections	*l* = −15→15

### Refinement

**Table:**

Refinement on *F*^2^	Primary atom site location: structure-invariant direct methods
Least-squares matrix: full	Secondary atom site location: difference Fourier map
*R*[*F*^2^ > 2σ(*F*^2^)] = 0.030	Hydrogen site location: inferred from neighbouring sites
*wR*(*F*^2^) = 0.068	H-atom parameters constrained
*S* = 1.06	*w* = 1/[σ^2^(*F*_o_^2^) + (0.0366*P*)^2^ + 4.1092*P*] where *P* = (*F*_o_^2^ + 2*F*_c_^2^)/3
2842 reflections	(Δ/σ)_max_ = 0.001
240 parameters	Δρ_max_ = 0.93 e Å^−^^3^
0 restraints	Δρ_min_ = −1.22 e Å^−^^3^

### Special details

**Table:**

Geometry. All e.s.d.'s (except the e.s.d. in the dihedral angle between two l.s. planes) are estimated using the full covariance matrix. The cell e.s.d.'s are taken into account individually in the estimation of e.s.d.'s in distances, angles and torsion angles; correlations between e.s.d.'s in cell parameters are only used when they are defined by crystal symmetry. An approximate (isotropic) treatment of cell e.s.d.'s is used for estimating e.s.d.'s involving l.s. planes.
Refinement. Refinement of *F*^2^ against ALL reflections. The weighted *R*-factor *wR* and goodness of fit *S* are based on *F*^2^, conventional *R*-factors *R* are based on *F*, with *F* set to zero for negative *F*^2^. The threshold expression of *F*^2^ > σ(*F*^2^) is used only for calculating *R*-factors(gt) *etc*. and is not relevant to the choice of reflections for refinement. *R*-factors based on *F*^2^ are statistically about twice as large as those based on *F*, and *R*- factors based on ALL data will be even larger.

### Fractional atomic coordinates and isotropic or equivalent isotropic displacement parameters (Å^2^)

**Table:**

	*x*	*y*	*z*	*U*_iso_*/*U*_eq_	Occ. (<1)
Dy	0.0000	0.612148 (16)	0.7500	0.02165 (10)	
N1	0.03494 (19)	0.6959 (2)	0.5922 (2)	0.0299 (7)	
N2	0.09455 (19)	0.7496 (2)	0.7881 (3)	0.0318 (8)	
N3	0.2176 (2)	0.3312 (2)	0.9692 (2)	0.0379 (9)	
H3	0.2412	0.2796	0.9869	0.046*	0.50
N4	0.16685 (19)	0.4740 (2)	1.0061 (2)	0.0295 (8)	
H4	0.1617	0.5157	1.0509	0.035*	
O1	0.09579 (16)	0.56623 (18)	0.88770 (19)	0.0302 (6)	
O2	0.07048 (16)	0.50051 (18)	0.68831 (19)	0.0327 (6)	
O3	0.15320 (18)	0.38990 (19)	0.6597 (2)	0.0362 (7)	
O4	0.2306 (2)	0.38078 (19)	1.1355 (2)	0.0437 (8)	
C1	0.1236 (2)	0.4394 (3)	0.7192 (3)	0.0278 (9)	
C2	0.1504 (2)	0.4274 (3)	0.8320 (3)	0.0262 (8)	
C3	0.1915 (3)	0.3504 (3)	0.8700 (3)	0.0363 (10)	
H3A	0.2025	0.3072	0.8224	0.044*	
C4	0.2065 (3)	0.3940 (3)	1.0424 (3)	0.0326 (9)	
C5	0.1350 (2)	0.4930 (2)	0.9059 (3)	0.0240 (8)	
C6	0.1293 (2)	0.7739 (3)	0.8822 (3)	0.0362 (10)	
H6	0.1307	0.7310	0.9350	0.043*	
C7	0.1639 (3)	0.8606 (3)	0.9064 (4)	0.0479 (12)	
H7	0.1875	0.8750	0.9736	0.057*	
C8	0.1626 (3)	0.9232 (3)	0.8298 (4)	0.0507 (13)	
H8	0.1847	0.9815	0.8448	0.061*	
C9	0.1283 (3)	0.9012 (3)	0.7289 (4)	0.0416 (11)	
C10	0.0959 (2)	0.8115 (3)	0.7112 (3)	0.0303 (9)	
C11	0.0644 (2)	0.7832 (3)	0.6071 (3)	0.0315 (9)	
C12	0.0658 (3)	0.8453 (3)	0.5253 (4)	0.0451 (11)	
C13	0.0962 (3)	0.9367 (4)	0.5480 (4)	0.0622 (15)	
H13	0.0954	0.9786	0.4945	0.075*	
C14	0.1256 (3)	0.9631 (3)	0.6439 (5)	0.0610 (15)	
H14	0.1449	1.0231	0.6559	0.073*	
C15	0.0367 (3)	0.8133 (4)	0.4259 (4)	0.0577 (14)	
H15	0.0352	0.8526	0.3699	0.069*	
C16	0.0108 (3)	0.7253 (4)	0.4111 (3)	0.0514 (13)	
H16	−0.0064	0.7029	0.3448	0.062*	
C17	0.0099 (2)	0.6686 (3)	0.4955 (3)	0.0384 (10)	
H17	−0.0091	0.6085	0.4839	0.046*	
O5	0.3087 (3)	0.3123 (3)	0.3401 (3)	0.0926 (15)	
H51	0.3201	0.2551	0.3440	0.139*	
H52	0.2760	0.3253	0.2854	0.139*	

### Atomic displacement parameters (Å^2^)

**Table:**

	*U*^11^	*U*^22^	*U*^33^	*U*^12^	*U*^13^	*U*^23^
Dy	0.02842 (15)	0.01487 (14)	0.01945 (14)	0.000	−0.00135 (10)	0.000
N1	0.0310 (18)	0.0264 (18)	0.0312 (18)	−0.0013 (14)	0.0023 (14)	−0.0005 (14)
N2	0.0311 (19)	0.0288 (19)	0.0350 (19)	−0.0034 (15)	0.0049 (15)	−0.0040 (15)
N3	0.060 (2)	0.0248 (18)	0.0264 (18)	0.0105 (17)	−0.0002 (16)	0.0024 (15)
N4	0.042 (2)	0.0245 (17)	0.0195 (16)	0.0066 (15)	0.0000 (14)	−0.0021 (13)
O1	0.0425 (16)	0.0218 (14)	0.0224 (13)	0.0096 (13)	−0.0044 (12)	−0.0024 (11)
O2	0.0439 (17)	0.0310 (15)	0.0209 (13)	0.0115 (13)	−0.0003 (12)	0.0015 (12)
O3	0.0519 (18)	0.0361 (16)	0.0211 (14)	0.0150 (14)	0.0079 (13)	−0.0015 (12)
O4	0.060 (2)	0.0342 (17)	0.0305 (16)	0.0059 (15)	−0.0095 (14)	0.0031 (13)
C1	0.036 (2)	0.021 (2)	0.025 (2)	−0.0020 (18)	0.0015 (17)	0.0014 (17)
C2	0.032 (2)	0.024 (2)	0.0217 (19)	0.0052 (17)	0.0024 (16)	0.0012 (16)
C3	0.053 (3)	0.025 (2)	0.029 (2)	0.010 (2)	0.0043 (19)	−0.0041 (18)
C4	0.039 (2)	0.028 (2)	0.028 (2)	−0.0024 (18)	−0.0028 (17)	0.0038 (17)
C5	0.029 (2)	0.021 (2)	0.0211 (19)	−0.0003 (16)	0.0017 (15)	0.0000 (15)
C6	0.038 (2)	0.033 (2)	0.035 (2)	−0.0083 (19)	0.0024 (19)	−0.0072 (19)
C7	0.043 (3)	0.048 (3)	0.052 (3)	−0.014 (2)	0.009 (2)	−0.024 (2)
C8	0.051 (3)	0.034 (3)	0.068 (3)	−0.016 (2)	0.013 (3)	−0.019 (3)
C9	0.042 (3)	0.024 (2)	0.061 (3)	−0.0076 (19)	0.014 (2)	−0.006 (2)
C10	0.025 (2)	0.026 (2)	0.040 (2)	−0.0013 (16)	0.0070 (17)	−0.0005 (18)
C11	0.029 (2)	0.029 (2)	0.037 (2)	0.0015 (17)	0.0071 (18)	0.0051 (18)
C12	0.044 (3)	0.041 (3)	0.052 (3)	−0.003 (2)	0.012 (2)	0.014 (2)
C13	0.077 (4)	0.044 (3)	0.066 (4)	−0.014 (3)	0.013 (3)	0.022 (3)
C14	0.070 (4)	0.028 (3)	0.088 (4)	−0.015 (2)	0.022 (3)	0.005 (3)
C15	0.067 (4)	0.066 (4)	0.038 (3)	−0.010 (3)	0.007 (2)	0.024 (3)
C16	0.056 (3)	0.070 (4)	0.028 (2)	−0.013 (3)	0.007 (2)	0.005 (2)
C17	0.037 (2)	0.042 (3)	0.036 (2)	−0.004 (2)	0.0056 (19)	−0.005 (2)
O5	0.142 (4)	0.047 (2)	0.070 (3)	0.003 (3)	−0.027 (3)	−0.010 (2)

### Geometric parameters (Å, °)

**Table:**

Dy—O2^i^	2.256 (3)	C2—C5	1.420 (5)
Dy—O2	2.256 (3)	C3—H3A	0.9300
Dy—O1^i^	2.313 (2)	C6—C7	1.398 (6)
Dy—O1	2.313 (2)	C6—H6	0.9300
Dy—N2	2.554 (3)	C7—C8	1.355 (7)
Dy—N2^i^	2.554 (3)	C7—H7	0.9300
Dy—N1^i^	2.576 (3)	C8—C9	1.392 (7)
Dy—N1	2.576 (3)	C8—H8	0.9300
N1—C17	1.331 (5)	C9—C10	1.412 (5)
N1—C11	1.360 (5)	C9—C14	1.431 (7)
N2—C6	1.324 (5)	C10—C11	1.442 (6)
N2—C10	1.358 (5)	C11—C12	1.408 (6)
N3—C3	1.335 (5)	C12—C15	1.397 (7)
N3—C4	1.364 (5)	C12—C13	1.430 (7)
N3—H3	0.8600	C13—C14	1.331 (8)
N4—C5	1.365 (5)	C13—H13	0.9300
N4—C4	1.382 (5)	C14—H14	0.9300
N4—H4	0.8600	C15—C16	1.349 (7)
O1—C5	1.253 (4)	C15—H15	0.9300
O2—C1	1.279 (4)	C16—C17	1.386 (6)
O3—C1	1.239 (5)	C16—H16	0.9300
O4—C4	1.239 (5)	C17—H17	0.9300
C1—C2	1.488 (5)	O5—H51	0.8483
C2—C3	1.363 (6)	O5—H52	0.8510
			
O2^i^—Dy—O2	88.73 (14)	C5—C2—C1	123.4 (3)
O2^i^—Dy—O1^i^	74.32 (9)	N3—C3—C2	126.1 (4)
O2—Dy—O1^i^	81.97 (10)	N3—C3—H3A	117.0
O2^i^—Dy—O1	81.97 (10)	C2—C3—H3A	117.0
O2—Dy—O1	74.32 (9)	O4—C4—N3	122.5 (4)
O1^i^—Dy—O1	146.67 (13)	O4—C4—N4	121.7 (4)
O2^i^—Dy—N2	147.90 (10)	N3—C4—N4	115.8 (3)
O2—Dy—N2	105.34 (10)	O1—C5—N4	117.4 (3)
O1^i^—Dy—N2	135.32 (10)	O1—C5—C2	126.3 (3)
O1—Dy—N2	74.62 (10)	N4—C5—C2	116.3 (3)
O2^i^—Dy—N2^i^	105.34 (10)	N2—C6—C7	123.4 (4)
O2—Dy—N2^i^	147.90 (10)	N2—C6—H6	118.3
O1^i^—Dy—N2^i^	74.62 (10)	C7—C6—H6	118.3
O1—Dy—N2^i^	135.32 (10)	C8—C7—C6	118.7 (4)
N2—Dy—N2^i^	77.95 (15)	C8—C7—H7	120.6
O2^i^—Dy—N1^i^	79.80 (10)	C6—C7—H7	120.6
O2—Dy—N1^i^	148.05 (10)	C7—C8—C9	120.6 (4)
O1^i^—Dy—N1^i^	122.34 (10)	C7—C8—H8	119.7
O1—Dy—N1^i^	74.60 (9)	C9—C8—H8	119.7
N2—Dy—N1^i^	73.07 (10)	C8—C9—C10	116.9 (4)
N2^i^—Dy—N1^i^	63.96 (10)	C8—C9—C14	123.8 (4)
O2^i^—Dy—N1	148.05 (10)	C10—C9—C14	119.3 (4)
O2—Dy—N1	79.80 (10)	N2—C10—C9	122.7 (4)
O1^i^—Dy—N1	74.60 (9)	N2—C10—C11	118.3 (3)
O1—Dy—N1	122.34 (10)	C9—C10—C11	119.0 (4)
N2—Dy—N1	63.96 (10)	N1—C11—C12	122.6 (4)
N2^i^—Dy—N1	73.07 (10)	N1—C11—C10	117.7 (3)
N1^i^—Dy—N1	123.98 (14)	C12—C11—C10	119.8 (4)
C17—N1—C11	117.3 (4)	C15—C12—C11	117.2 (4)
C17—N1—Dy	123.9 (3)	C15—C12—C13	123.8 (4)
C11—N1—Dy	117.0 (2)	C11—C12—C13	118.9 (4)
C6—N2—C10	117.6 (3)	C14—C13—C12	121.6 (5)
C6—N2—Dy	123.4 (3)	C14—C13—H13	119.2
C10—N2—Dy	117.5 (2)	C12—C13—H13	119.2
C3—N3—C4	119.6 (3)	C13—C14—C9	121.3 (5)
C3—N3—H3	120.2	C13—C14—H14	119.3
C4—N3—H3	120.2	C9—C14—H14	119.3
C5—N4—C4	126.0 (3)	C16—C15—C12	120.0 (4)
C5—N4—H4	117.0	C16—C15—H15	120.0
C4—N4—H4	117.0	C12—C15—H15	120.0
C5—O1—Dy	132.2 (2)	C15—C16—C17	119.4 (4)
C1—O2—Dy	140.8 (2)	C15—C16—H16	120.3
O3—C1—O2	123.1 (3)	C17—C16—H16	120.3
O3—C1—C2	118.8 (3)	N1—C17—C16	123.3 (4)
O2—C1—C2	118.1 (3)	N1—C17—H17	118.3
C3—C2—C5	116.1 (3)	C16—C17—H17	118.3
C3—C2—C1	120.5 (3)	H51—O5—H52	112.0
			
O2^i^—Dy—N1—C17	−6.9 (4)	C1—C2—C3—N3	−178.9 (4)
O2—Dy—N1—C17	63.8 (3)	C3—N3—C4—O4	−178.8 (4)
O1^i^—Dy—N1—C17	−20.6 (3)	C3—N3—C4—N4	1.0 (6)
O1—Dy—N1—C17	127.8 (3)	C5—N4—C4—O4	−176.8 (4)
N2—Dy—N1—C17	176.6 (3)	C5—N4—C4—N3	3.4 (6)
N2^i^—Dy—N1—C17	−98.8 (3)	Dy—O1—C5—N4	−158.4 (3)
N1^i^—Dy—N1—C17	−139.5 (3)	Dy—O1—C5—C2	22.1 (6)
O2^i^—Dy—N1—C11	157.4 (2)	C4—N4—C5—O1	174.7 (4)
O2—Dy—N1—C11	−132.0 (3)	C4—N4—C5—C2	−5.7 (6)
O1^i^—Dy—N1—C11	143.6 (3)	C3—C2—C5—O1	−177.0 (4)
O1—Dy—N1—C11	−68.0 (3)	C1—C2—C5—O1	2.6 (6)
N2—Dy—N1—C11	−19.2 (3)	C3—C2—C5—N4	3.4 (5)
N2^i^—Dy—N1—C11	65.4 (3)	C1—C2—C5—N4	−177.0 (3)
N1^i^—Dy—N1—C11	24.7 (2)	C10—N2—C6—C7	2.7 (6)
O2^i^—Dy—N2—C6	8.4 (4)	Dy—N2—C6—C7	−162.9 (3)
O2—Dy—N2—C6	−104.8 (3)	N2—C6—C7—C8	−0.4 (7)
O1^i^—Dy—N2—C6	161.1 (3)	C6—C7—C8—C9	−0.9 (7)
O1—Dy—N2—C6	−36.2 (3)	C7—C8—C9—C10	−0.2 (7)
N2^i^—Dy—N2—C6	108.1 (3)	C7—C8—C9—C14	−179.1 (5)
N1^i^—Dy—N2—C6	42.0 (3)	C6—N2—C10—C9	−3.9 (6)
N1—Dy—N2—C6	−175.0 (3)	Dy—N2—C10—C9	162.6 (3)
O2^i^—Dy—N2—C10	−157.3 (3)	C6—N2—C10—C11	174.8 (4)
O2—Dy—N2—C10	89.6 (3)	Dy—N2—C10—C11	−18.7 (5)
O1^i^—Dy—N2—C10	−4.5 (3)	C8—C9—C10—N2	2.7 (6)
O1—Dy—N2—C10	158.1 (3)	C14—C9—C10—N2	−178.4 (4)
N2^i^—Dy—N2—C10	−57.6 (2)	C8—C9—C10—C11	−176.0 (4)
N1^i^—Dy—N2—C10	−123.7 (3)	C14—C9—C10—C11	2.9 (6)
N1—Dy—N2—C10	19.3 (3)	C17—N1—C11—C12	2.9 (6)
O2^i^—Dy—O1—C5	68.0 (3)	Dy—N1—C11—C12	−162.4 (3)
O2—Dy—O1—C5	−22.9 (3)	C17—N1—C11—C10	−176.5 (4)
O1^i^—Dy—O1—C5	23.4 (3)	Dy—N1—C11—C10	18.3 (4)
N2—Dy—O1—C5	−134.2 (4)	N2—C10—C11—N1	0.1 (5)
N2^i^—Dy—O1—C5	171.7 (3)	C9—C10—C11—N1	178.8 (4)
N1^i^—Dy—O1—C5	149.6 (4)	N2—C10—C11—C12	−179.3 (4)
N1—Dy—O1—C5	−89.6 (3)	C9—C10—C11—C12	−0.6 (6)
O2^i^—Dy—O2—C1	−72.1 (4)	N1—C11—C12—C15	−1.3 (7)
O1^i^—Dy—O2—C1	−146.4 (4)	C10—C11—C12—C15	178.1 (4)
O1—Dy—O2—C1	9.9 (4)	N1—C11—C12—C13	178.6 (4)
N2—Dy—O2—C1	78.7 (4)	C10—C11—C12—C13	−2.0 (6)
N2^i^—Dy—O2—C1	170.4 (4)	C15—C12—C13—C14	−177.8 (6)
N1^i^—Dy—O2—C1	−3.8 (5)	C11—C12—C13—C14	2.3 (8)
N1—Dy—O2—C1	137.9 (4)	C12—C13—C14—C9	0.1 (9)
Dy—O2—C1—O3	−176.1 (3)	C8—C9—C14—C13	176.1 (5)
Dy—O2—C1—C2	4.2 (6)	C10—C9—C14—C13	−2.8 (8)
O3—C1—C2—C3	−15.5 (6)	C11—C12—C15—C16	−1.7 (8)
O2—C1—C2—C3	164.1 (4)	C13—C12—C15—C16	178.4 (5)
O3—C1—C2—C5	164.9 (4)	C12—C15—C16—C17	2.9 (8)
O2—C1—C2—C5	−15.4 (6)	C11—N1—C17—C16	−1.7 (6)
C4—N3—C3—C2	−3.0 (7)	Dy—N1—C17—C16	162.5 (3)
C5—C2—C3—N3	0.7 (7)	C15—C16—C17—N1	−1.2 (8)

Symmetry codes: (i) −*x*, *y*, −*z*+3/2.

### Hydrogen-bond geometry (Å, °)

**Table:**

*D*—H···*A*	*D*—H	H···*A*	*D*···*A*	*D*—H···*A*
N3—H3···N3^ii^	0.86	1.80	2.659 (7)	174
N4—H4···O3^iii^	0.86	2.01	2.867 (4)	178
N4—H4···O2^iii^	0.86	2.62	3.183 (4)	124
O5—H51···O3^iv^	0.85	2.15	2.993 (5)	175
O5—H52···O4^v^	0.85	2.15	2.960 (5)	160

Symmetry codes: (ii) −*x*+1/2, −*y*+1/2, −*z*+2; (iii) *x*, −*y*+1, *z*+1/2; (iv) −*x*+1/2, −*y*+1/2, −*z*+1; (v) *x*, *y*, *z*−1.
